# 

**Published:** 1809-01

**Authors:** 


*LONDON:*

PRINTED FOR RICHARD PHILLIPS, NO. 6, BRIDGE-STREET, BLACKFRIARS.

### NEW MEDICAL PUBLICATIONS.

A Treatise on Scrophula. By James Russell, Fellow of the Royal College of Surgeons, and Professor of Clinical Surgery in the University of Edinburgh. 8vo. 5s.

The London Medical Dictionary. By Bartholomew Pars, Fellow of the Royal Societies of London and Edinburgh, and senior Physician of the Exeter Hospital. 2 vols 4to. 4l. 10s. boards.

Cases of Diabetes, Consumption, &c. with Observations on the History and Treatment of Diseases in general. By Robert Watt, Member of the Faculty of Physicians and Surgeons, Glasgow. 8vo. 8s.

### To CORRESPONDENTS.

We received Mr. HARRUP's remarks on our promised Index, with a full conviction of his good intentions. The Index has been in a courſe of preparation by Dr. BRADLEY from the time it was firſt announced, and will certainly follow, with all poſſible ſpeed, after the appearance of our twenty-fourth volume.

We ſhall be much obliged to Dr. MOSSMAN to reviſe his paper, as it is conſiderably too long for inſertion. If Dr. M. finds it requiſite to take notice of any obſervations contained in our Journal, we could wiſh he would do it without any unneceſſary references to other writers.

Mr. CHRISTIE's Account of Vaccination in Ceylon during the year 1807, will appear in our next Number.

Mr. HARRUP's Communication ſhall alſo appear in our next.

